# Characterization of facultative yet endohyphal interaction between *Umbelopsis* and *Paraburkholderia*

**DOI:** 10.1186/s12864-026-13055-5

**Published:** 2026-06-19

**Authors:** Alicja Okrasińska, Maria Furman, Jakub Lisiak, Anna Michalik, Julia Pawłowska

**Affiliations:** 1 Faculty of Biology, Institute of Evolutionary Biology, Biological and Chemical Research Centre, University of Warsaw, Warsaw, Poland; 2 Faculty of Biology, Institute of Zoology and Biomedical Research, Department of Developmental Biology, Jagiellonian University, Cracow, Poland

**Keywords:** Bacterial-fungal interactions, Endohyphal bacteria, *Mucoromycota*, *Burkholderiaceae*, Symbiosis, Deadwood

## Abstract

**Background:**

The generalist and facultative symbioses are more common in nature and usually precede obligate and specialized ones. Although bacterial-fungal interactions are probably not an exception, little is known about the role that facultative bacterial-fungal symbioses may play in adaptation to occupying specific niches and changing environmental conditions. While *Mucoromycota* fungi are known to form close relationships with endohyphal bacteria, examples of more facultative interactions have been recently reported in this lineage as well. One example of facultative, yet endohyphal interaction is *Umbelopsis-Paraburkholderia* consortium which seems to be highly prevalent in deadwood. In this study we aimed to characterize this relationship as, so far, in-depth characterizations are available only for strictly endohyphal bacteria and their hosts.

**Results:**

We compared functionally annotated genomes of both partners with genomes of their free-living and symbiotic relatives. Analysis showed that the genome of *Paraburkholderia* does not differ greatly from genomes of free-living *Burkholderiaceae*, as it is not reduced and possesses a similar enzymatic potential. The genome of the fungal partner is also similar to the genomes of its close relatives, however *Umbelopsis* belongs to a relatively understudied taxonomic group, thus conclusions about its genomic content are less certain. In the genomes we identified some features needed for establishment and maintenance of endohyphal interaction, such as genes coding for diacylglycerol kinase (DGK) and secretion systems. Finally, we detected potential metabolic intertwinement in wood degrading capacities of partners, which, combined with the detection of genes encoding plant growth promotion factors in bacteria, allows us to assume that the consortium is well adapted to develop in the decaying wood ecosystem from which it was isolated.

**Conclusions:**

While presence of *Paraburkholderia* decreases growth rate of *Umbelopsis* in the simplified laboratory system, it is possible that together they may occupy niches not available to each partner individually. *Paraburkholderia*, unlike strictly endohyphal symbionts, has a non-reduced genome, with extensive metabolic capacities which probably play an important role in adaptation to colonizing otherwise inaccessible niches by their fungal host.

**Supplementary Information:**

The online version contains supplementary material available at https://doi.org/10.1186/s12864-026-13055-5.

## Background

Bacteria and fungi can often be found coexisting in the same niche. Even though both groups face the same challenges in specific habitats, they have different characteristics and abilities. In non-homogeneous substrates, such as soil, one of the biggest challenges for microorganisms is to get from one nutrient-rich microniche to another. While some bacteria have flagella or other movement organelles, filamentous fungi can quickly colonize much bigger areas, due to their hyphal growth. There are bacteria known to use hyphae as so-called “fungal highways” to colonize otherwise unreachable areas [1]. Moreover, as hyphae grow, the area around them becomes less aerated which provides good environmental conditions for soil-dwelling anaerobic bacteria [2]. On the other hand, bacteria can expand the metabolic capabilities of fungi, e.g. by producing toxins allowing for colonization of a plant [3] or providing essential nutrients by fixing nitrogen [4]. These differences often complement each other making fungi and bacteria prone to interact.

These bacterial-fungal interactions (abbrev. BFI) vary in strictness and spatial closeness. The least strict type would be an opportunistic soil-dwelling bacteria that attaches to any hyphae in the soil to travel to yet uncolonized niche. This kind of epihyphal interaction is usually spontaneous and ephemeral. The most intimate however is when prokaryotic symbionts are endohyphal, i.e., live within the hyphae, forming a stable holobiont in an evolutionary time scale. Although probably spontaneous and transient interactions are much more common in nature [5], the strictest, coevolving interactions have been most thoroughly studied.

So far, most in-depth studies on BFI focused on interactions between *Mucoromycota* representatives and two groups of endohyphal bacteria (abbrev. EHB): *Burkholderiaceae*-related endosymbionts (abbrev. BRE) and *Mycoplasma-*related endosymbionts (or *Mollicutes*-related endosymbionts; abbrev. MRE) [6]. However, a deeper functional understanding of the symbiosis between fungi and associated bacteria is available only for a few examples of BRE. While physiology and molecular mechanisms of these interactions remain elusive, it seems that lipid metabolism seems to play an important role in all of them.

*Mycetohabitans* sp., now a model for studying EHB, allows its host *Rhizopus microsporus* to parasitize rice seedlings at the cost of total control of its asexual reproduction and partial control over sexual reproduction [7, 8]. While *Mycetohabitans* sp. symbionts seem to be coevolving with their host, they remain cultivable, unlike other BRE and MRE. Recently it has been also proven that EHB protect *Rhizopus* against micropredators [9]. In another example, *Mycoavidus cysteinexigens* was shown to slow the growth of *Linnemannia elongata* and to be unable to grow on own [10]. Analysis of the reduced genome of the bacterium suggested that it is unable to biosynthesize several essential amino acids, which are provided by the fungal host [10]. It was also suggested by the same authors that the expansion of *fadD* genes in the endohyphal bacteria genome enables it to metabolize fatty acids produced by its fungal host.

Endohyphal *Candidatus* Glomeribacter gigasporarum helps arbuscular mycorrhizal fungi from the *Gigasporales* order form relationships with host plants by increasing ATP production and inducing detoxification of reactive oxygen forms [11]. It was also observed that the spores of arbuscular mycorrhizal fungus devoid of these EHB had less lipid droplets than the ones with the endosymbionts [12]. Genome analysis of the host fungus showed that it lacks genes encoding the fatty acid synthase enzyme complex and thus, is unable to produce palmitic acid without either EHB or a symbiotic plant host [13]. All these examples show that EHB can greatly impact their host’s metabolism even if their genomes are usually reduced in size [6, 14].

In all of the abovementioned pairs, bacteria are endohyphal, the relationships are obligatory for bacterial partners, and they coevolve with fungal hosts. It is however important to remember that all of these characteristics: endohyphality, facultativeness, and evolutionary stability are spectra and not binary categories. This is one of the reasons why it is only recently that attention was also paid to more facultative interactions between free-living, possibly epihyphal bacteria and fungi [5, 15, 16]. While Tláskal et al. [16] showed functionally complementary roles of fungal and bacterial communities in deadwood decomposition, Telagathoti et al. [15] reported non-random associations between previously overlooked epihyphal bacteria and *Mortierellomycotina* fungi, suggesting their potential ecological significance.

In this study, we focused on the interactions formed by another representative of *Mucoromycota*, *Umbelopsis.* The ecology of *Umbelopsidales* is relatively understudied compared to obligate plant symbionts from *Endogonales* and saprotrophic *Mucorales*, both of which are considered sister clades to *Umbelopsidales* in most recent phylogenetic studies [17]*.* Fungi from this group are commonly isolated not only from soil [18] but also from the rhizosphere [19, 20], decomposing wood [21–23], and as stem [24, 25] and root [26, 27] endophytes. While their presence in these niches and biomes is clearly established, their ecological role has not been studied thoroughly.

Recent screening of *Umbelopsis* strains revealed a high prevalence of associated bacteria from predominantly free-living *Paraburkholderia* genus (abbrev. PRE) [28]. Closely related *Paraburkholderia* representatives were also detected in *Mortierellomycotina*, other *Mucoromycotina*, and *Gigasporaceae* representatives [28, 29], however it is not clear whether they are epihyphal or endohyphal. Although interaction between *Umbelopsis* and *Paraburkholderia* seems to be facultative and there are no signs of coevolution between partners [28], we speculate that it might represent an intermediate stage between free-living epihyphal bacterial communities and intimate endohyphal relationships such as ones with BRE and MRE. While current state of knowledge does not allow for understanding the evolutionary position of *Umbelopsis-Paraburkholderia* in the context of other bacterial-fungal partnerships, in this article we aim to provide genomic insights on functioning of this facultative yet endohyphal relationship. To this end, we analyze the genomes of both partners forming this interaction, using genomes of closely related symbiotic and free-living organisms as a reference background.

## Methods

### Study objects

*Umbelopsis* strain WA0000050703 with naturally co-occurring *Paraburkholderia* strain KKMWBUW1 was isolated from dead spruce (*Picea abies*) bark from Białowieża Primeval Forest (Podlaskie Voivodeship, Poland) in October 2018. The consortium was maintained on potato dextrose agar medium (PDA) at room temperature. Axenic fungal culture was obtained by passaging the consortium via PDA with the addition of antibiotics (100 µg/mL ciprofloxacin, 50 µg/mL kanamycin, 50 µg/mL streptomycin, 50 µg/mL chloramphenicol). After three passages, the axenicity of the culture was confirmed by amplification of 16S rRNA bacterial fragments and subsequent electrophoresis. As according to our previous observations (data not shown) increased temperatures often permit bacteria to overgrow fungi, axenic bacterial culture was obtained by reduction plating from the initial consortium plate at 30 °C.

### Microscopic evaluation of the consortium

Fragments of *Umbelopsis-Paraburkholderia* consortium (EHB +) hyphae were preserved in 2.5% glutaraldehyde in 0.1 M phosphate buffer. After one day of fixation, the material was washed in a phosphate buffer with addition of sucrose (5,8 g/100 ml) and then postfixed in 1% tetroxide osmium. Next, samples were dehydrated in the graded series of ethanol (30–100%) and acetone and embedded in epoxy resin Epon 812 (SERVA, Heidelberg, Germany). Ultrathin Sects. (90 nm thick) were contrasted with uranyl acetate and lead citrate and examined and photographed in Jeol JEM 2100 electron transmission microscope (TEM) at 80 kV.

### Comparative growth essays

To assess the impact of bacteria on fungal growth, a co-culture experiment was performed. *Umbelopsis-Paraburkholderia* consortium (EHB +, control) and *Umbelopsis* cured of bacteria (EHB-, experiment) were plated on PDA. After a week, spores from the colonies were collected and suspended in 0.9% saline solution. Absorbance of the spore solutions were measured using SpectroStar nano. Then, based on absorbance and previous calculations of number of spores, appx. 5 × 10^5^ spores were added to flasks with either 1) mineral liquid medium with 0.4% glucose (NH_4_NO_3_ 3 g/L, KH_2_PO_4_ 1 g/L, MgSO_4_ × 7H_2_O 0.5 g/L, KCl 0.5 g/L, glucose 0.4 g/L), or 2) rich medium (Potato Dextrose Broth). The flasks were kept at 25 °C and 100 rpm for 10 to 14 days. Every 12 or 24 h mycelium from a randomly chosen flask was centrifuged, dried in 105 °C for 72 h and then weighed. Each variant of the experiment (EHB + in rich medium, EHB + in minimal medium, EHB- in rich medium, EHB- in minimal medium) had three biological replicates. Data from all replicates were then used to prepare growth graphs for *Umbelopsis* with and without *Paraburkholderia* using ggplot2 v3.5.2 [30] in R v4.4.1 [31] in RStudio v2024.04.2 [32].

### DNA extraction and sequencing

Whole genomic DNA was extracted from fungus and bacteria separately using EURx GeneMatrix Bacterial & Yeast Genomic DNA Purification Kit according to manufacturer’s protocol with an additional preparation step of grinding material in liquid nitrogen for the fungus. Both extracts were quality checked on the NanoPhotometer NP80 (Implen) and Qubit 3.0 Fluorometer (Invitrogen) instruments. Additionally, for measuring DNA fragmentation for MinION Nanopore sequencing, TapeStation 4150 (Agilent) was also used.

Libraries for Illumina sequencing were prepared and sequenced by an external company (Genomed SA). Fungal genome was sequenced in paired-end reads mode (2 × 150 bp) on Illumina NovaSeq instrument, and bacterial genome in paired-end reads mode (2 × 300 bp) on Illumina MiSeq.

Libraries for long read sequencing from both fungus and bacteria were prepared using Ligation Sequencing Kit SQK-LSK109 (Oxford Nanopore) according to the manufacturer’s protocol. The libraries were then sequenced on Flow Cell R9.4.1 primed with Flow Cell Priming Kit EXP-FLP002 on MinION Nanopore instrument (Oxford Nanopore).

### RNA extraction and sequencing

RNA was extracted from two samples of the consortium grown in rich liquid medium (PDB, potato dextrose broth) at 25 °C. After a week after inoculation, the medium was discarded and the biomass from two flasks were used in subsequent steps. TRIzol reagent was added to the tube with biomass (1 mL per 100 mg of mycelium) and quickly vortexed. After 15 min of RT incubation, chloroform:isoamyl 24:1 was added to each sample (0.2 mL per 1 mL of TRIzol). After two-minute vortexing, five-minute room temperature incubation, and centrifugation (4 °C, 15 min, 12,000 g), aqueous phase was carefully transferred into a clean Eppendorf tube. RNA was precipitated by mixing the water phase with an equal amount of isopropanol, 10-min RT incubation and centrifuged as in the previous step. After discarding the supernatant, RNA pellets were dried, washed twice with 70% ethanol, and resuspended in PCR-grade water. Any residual DNA contamination was removed by using DNase TURBO (Invitrogen, USA) according to the manufacturer’s protocol. Then, RNA was purified using GeneJET RNA Cleanup and Concentration Micro Kit (Thermo Scientific, USA). Quantity and quality of obtained RNA was verified using the same methods as described for DNA.

RNA-seq libraries were prepared by the Genomic Core Facility in the Centre of New Technologies (University of Warsaw) with the usage of RiboFree method. Libraries were then sequenced on Illumina NovaSeq platform in paired-end reads mode (2 × 100 bp).

### Genomic and transcriptomic bioinformatic analysis

MinION Nanopore DNA reads obtained from fungal culture were basecalled using Guppy v.6.0.1 (https://community.nanoporetech.com) with default settings and the ones that passed guppy, were then quality checked using pycoQC v.2.5.2 [33]. Illumina NovaSeq fungal genomic reads were quality checked and visually inspected using FastQC v012.1 [34]. Nanopore reads were then assembled using flye v2.9.2 [35] and polished with Illumina reads using polypolish v0.5.0 [36]. Assembly was evaluated using Quast v5.2.0 [37] and its completeness was verified by BUSCO v5.8.2 [38].

Initial bacterial assemblies were created with Unicycler v0.5.1 [39] using both long and short reads without any subsequent polishing, as suggested by its developers. Similarly as for fungus, Quast v5.2.0 [37] and BUSCO v5.8.2 [38] were used to evaluate the assembly.

Raw RNA-seq reads obtained from the consortium were mapped to the assemblies using hisat2 v2.2.1 [40], and separated into bacterial and fungal datasets, after removal of reads assigned to both. Fungal dataset was then used as an aid for gene calling step in funannotate pipeline, while bacterial dataset was not used in present study.

### Genome annotation

Before gene prediction and annotation was performed using Funannotate v1.8.15 [41], the fungal genome assembly was prepped as suggested by its developers. Prep included cleaning redundant contigs using minimap2 v.2.26 [42], sorting, and softmasking repeats using tantan v40 [43]. Training gene predictors (funannotate train function) was aided by adding two sets of fungal RNA-seq data. The same gene calling and annotation steps were performed for all the other fungal genomes analyzed in this study (Table 1). Bakta v1.10.3 [44] was used for gene calling and initial functional annotation of the bacterial genome. Bakta was also used for gene calling and annotation for all the bacterial strains used as a comparison database (Table 2).

**Table 1:** Basic characteristics of the fungal genomes used in this study

Species	Strain	Source	Assembly accession	Genome size (Mb)	Contigs	%GC	Genes	Proteins	tRNA
*Mortierella alpina*	CGMCC 20262	soil	GCA_029449245.1	40.22	28	50.92%	11,321	11,100	221
*Lobosporangium transversale*	NRRL-3116	soil	GCA_002105155.1	42.20	138	41.57%	11,677	11,519	158
*Umbelopsis* sp.	PMI-123	poplar rhizosphere	GCA_021235465.1	24.64	38	41.50%	9195	9085	110
*Umbelopsis ramanniana*	AG	no data	GCA_025399195.1	23.08	198	43.13%	8951	8827	124
*Umbelopsis* sp.	AD052	no data	GCA_025677805.1	22.50	67	43.07%	8710	8602	108
*Umbelopsis vinacea*	WA0000051536	soil	GCA_016758895.1	23.15	150	43.09%	8785	8651	134
***Umbelopsis sp.***	**WA0000050703**	**decaying spruce**	**submitted**	**23.01**	**69**	**42.08%**	**9194**	**9040**	**154**
*Umbelopsis isabellina*	NBRC-7884	no data	GCA_000534915.1	22.59	84	41.79%	8720	8647	73
*Umbelopsis* sp.	M5902	human feces	GCA_037039815.1	22.01	58	42.17%	8457	8330	127
*Umbelopsis isabellina*	WA0000067209	soil	GCA_016758905.1	22.05	64	41.99%	8659	8530	129
*Umbelopsis isabellina*	B7317	human body	GCA_000697415.1	21.86	58	41.98%	8654	8535	119
*Umbelopsis isabellina*	MPG-14A	no data	GCA_027595865.1	21.84	184	41.95%	8731	8604	127

Bold indicates the strain from the studied here consortium

**Table 2:** Basic characteristics of the bacterial genomes used in this study

Species	Strain	Source/host	Assembly accession	EHB	Genome size (Mb)	Contigs	GC (%)	CDS	Coding density	rRNAs	tRNAs	ANI (%)
*Glomeribacter gigasporarum*	BEG34	*Gigaspora margarita*	GCF_000227585.1	yes	1.73	120	54.8	1935	87.0	3	41	70.5
*Mycoavidus cysteinexigens*	B1EB	*Linnemannia elongata*	GCF_003966915.1	yes	2.80	1	48.9	2377	88.6	6	45	69.3
*Mycoavidus cysteinexigens*	HKI	*Podila verticillata*	GCF_020023735.2	yes	2.25	1	50.7	1766	91.2	6	43	69.9
*Mycoavidus* sp.	SF9855	*Podila verticillata*	GCF_024582735.1	yes	2.23	1	50.3	1792	90.7	6	43	69.5
*Mycetohabitans rhizoxinica*	HKI454	*Rhizopus microsporus*	GCF_000198775.1	yes	3.75	3	60.7	3331	84.9	9	49	74.8
*Mycetohabitans* sp.	B8	*Rhizopus microsporus*	GCF_021991875.1	yes	3.28	34	61.2	2875	84.2	3	44	74.5
*Mycetohabitans endofungorum*	HKI456	*Rhizopus microsporus*	GCF_002927045.1	yes	3.29	76	61.3	2978	82.8	3	45	75.1
*Mycetohabitans* sp.	B5	*Rhizopus microsporus*	GCF_021991855.1	yes	3.30	73	61.3	2974	82.6	3	46	75.0
*Paraburkholderia tropica*	P-31	pomegranate rhizosphere	GCF_001673675.1	no	8.91	148	64.7	7745	85.6	3	49	77.9
*Paraburkholderia bryophila*	H2C3B	no data	GCF_013408935.1	no	8.31	2	62.7	7530	86.3	21	65	84.7
*Paraburkholderia phytofirmans*	PsJN	onion roots	GCF_000020125.1	no	8.21	3	62.3	7336	86.2	18	65	85.4
*Paraburkholderia fungorum*	ATCC BAA-463	*Phanerochaete chrysosporium*	GCF_000961515.1	no	9.06	4	61.8	8175	86.3	18	64	88.1
*Paraburkholderia* sp.	OLGA172	spruce forest soil	GCF_001634365.1	no	8.57	5	60.9	7781	84.2	21	65	90.0
*Paraburkholderia caffeinilytica*	CF1	tea rhizosphere	GCF_003368325.1	no	8.32	3	62.2	7342	86.2	18	69	90.9
***Paraburkholderia* sp.**	**KKMWBUW1**	***Umbelopsis* sp.**	**submitted**	**yes**	**9.06**	**3**	**61.3**	**8085**	**86.6**	**18**	**68**	**100**
*Paraburkholderia aspalathi*	SEWS4	soil	GCF_026309755.1	no	8.17	50	61.5	7280	87.3	3	48	91.0
*Paraburkholderia madseniana*	RP11	forest soil	GCF_009690905.1	no	10.09	284	61.4	9223	84.2	6	56	91.2
*Janthinobacterium tructae*	SNU-WT1	rainbow trout	GCF_006517255.1	no	6.31	1	62.4	5428	90.4	25	94	-
*Janthinobacterium lividum*	EIF2	water pond	GCF_013372065.1	no	6.76	2	62.4	5856	90.1	25	94	-

Bold indicates the strain from the studied here consortium

Carbohydrate-active enzymes (CAZymes) in predicted proteomes of both bacterium and fungus were identified using the run_dbcan3 pipeline [45] with default settings. As suggested by the authors of the pipeline, we decided to use only these CAZyme predictions which were identified by at least two of the three tools used (HMMER run versus dbCAN database, DIAMOND blast against CAZy database, HMMER run versus dbCAN-sub database).

Predicted proteomes of both fungus and bacteria were mapped onto the KEGG database [46] using KEGG Automatic Annotation Server (KAAS) [47]. The KAAS analysis was run using bi-directional best hit method to assign orthologs with appropriate "for Eukaryotes" and "for Prokaryotes" representative gene sets as a reference. Obtained KEGG Orthology (KO) assignments were combined into a single dataset within which each KO has been annotated with one of the following categories: unique to fungus, unique to bacteria, shared. The dataset was uploaded to KEGG Mapper Color, and each of the available metabolic maps was investigated for potential co-metabolism between the partners.

In order to confirm the presence of genes encoding particular enzymes, a local BLAST protein database from the predicted proteomes of all analyzed fungal and bacterial strains was created. The blastp algorithm v2.14.0 [48] was used to verify the presence of homologs of the genes in question in the genomes of the organisms in the database. Hit was considered viable if E-value < 1e-30, and there was over 40% of identities. TXSS software [49] was used for all bacterial proteomes to verify the presence of proteins building each type of secretion system. Specifically, for assessing the presence of the type VI secretion system (T6SS) proteins, which enable bacteria to interact with their adjacent cells, local database was queried with experimentally confirmed T6SS proteins from *Paraburkholderia phymatum* downloaded from SecReT6 [50].

### Phylogenetic placement of the isolates

All publicly available genomes of *Umbelopsis* strains and two *Mortierellaceae* strains were downloaded from the NCBI database and prepped using funannotate as described in the previous section. Then, proteomes predicted by funannotate and used as an input for Orthofinder v3.0.1b1 [51] phylogenomic analysis. Within Orthofinder, orthogroups were found, and then, single-copy orthogroups were aligned by MAFFT [52]. Gene alignments were then concatenated and FastTree [53] and STRIDE [54] were used to calculate and root the species tree. Obtained tree was then annotated and visually edited using iTOL [55].

To determine genetic relatedness between endohyphal *Paraburkholderia* strain KKMWBUW1 with other closely related taxa, ANIclustermap wrapper script (https://github.com/moshi4/ANIclustermap) with skani algorithm [56] was used. Proteomes predicted from genomes of the same bacteria were also used for phylogenomic analysis using Orthofinder. SH matching analysis tool [57] was used to verify whether ITS sequence of *Umbelopsis* WA0000050703 belongs to any of named or unnamed SH clusters present in the UNITE database.

## Results

### Growth in axenic and non-axenic conditions

After successfully curing *Umbelopsis* of *Paraburkholderia*, we compared the growth rate of cured (EHB-) and wildtype (EHB +) *Umbelopsis.* Growth essays showed that bacteria slow fungal growth in the minimal medium. Similar observation of stunted growth of *Umbelopsis* in presence of *Paraburkholderia* was made when organisms were grown on rich medium – potato dextrose broth (PDB). Growth measurements for both conditions are shown in Additional file 1, Fig. AF1, while raw data used for the plots is in Additional file 2. As repassaging was done by collecting spores and using spore suspension as the inoculum, which did not cause loss of EHB (also confirmed by amplification of 16S rRNA gene and subsequent electrophoresis), bacteria must be associated with spores and thus, vertically transmitted.

*Paraburkholderia* was also able to grow axenically on both rich standard mycological medium (PDB) and minimal mineral medium (see Methods), which further proves non-obligatory nature of this relationship for both partners.

### Visualization of bacteria within fungal hyphae

**Fig. 1:**
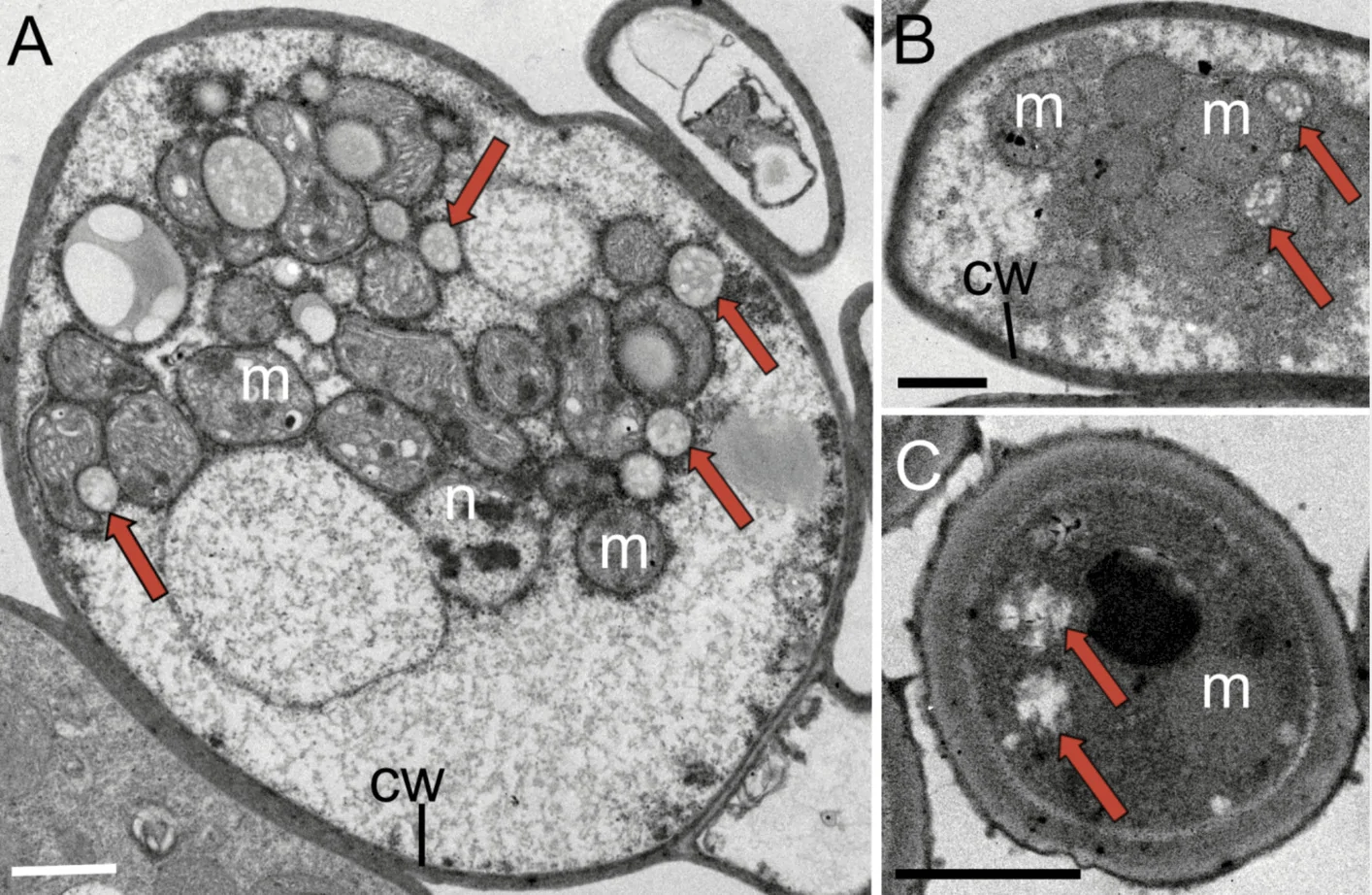
Bacterial cells inside hyphal cytoplasm (**A**, **B**), and spore (**C**) of *Umbelopsis* sp. WA0000050703 visualized using transmission electron microscope. Bacterial cells are indicated with arrows, cw – cell wall, m – mitochondrion, n – nucleus, scale bar = 1 µm

Ultrastructural analysis of *Umbelopsis* hyphae revealed that their cells are surrounded by a thick cell wall followed by a plasma membrane. The cytoplasm is filled with numerous large mitochondria with a complex system of internal membranes. A prominent, spherical nucleus is present in each cell, containing multiple nucleoli. *Paraburkholderia* cells were distributed throughout the hyphae cytoplasm, located among the mitochondria. Their cytoplasm contains numerous clearly visible, electron-light granules (Fig. 1A, B). The *Paraburkholderia* cells were also observed in the cytoplasm of *Umbelopsis* spores (Fig. 1C).

### Basic characteristics of the *Umbelopsis* genome

DNA extracted from cured *Umbelopsis* was sequenced using both long-read and short-read technologies. From MinION Nanopore sequencing, 207 778 reads were obtained with median length of 3880 bases and median PHRED score of 8.77, which resulted in 1 181 450 000 bases sequenced. After guppy basecalling, 147 592 reads (ca. 71%) were retained with a median length of 4030 bases and median PHRED score of 10.04, which resulted in 872 102 200 (ca. 74%) bases passing the basecalling process. Illumina NovaSeq sequencing resulted in 11 994 243 paired-end reads, all of them uncontaminated and of very good quality (median PHRED score per base above 34 for all bases), therefore no additional cleaning steps were performed and all reads were retained. Gene calling was aided by two sets of RNA-seq data (see Methods).

**Fig. 2:**
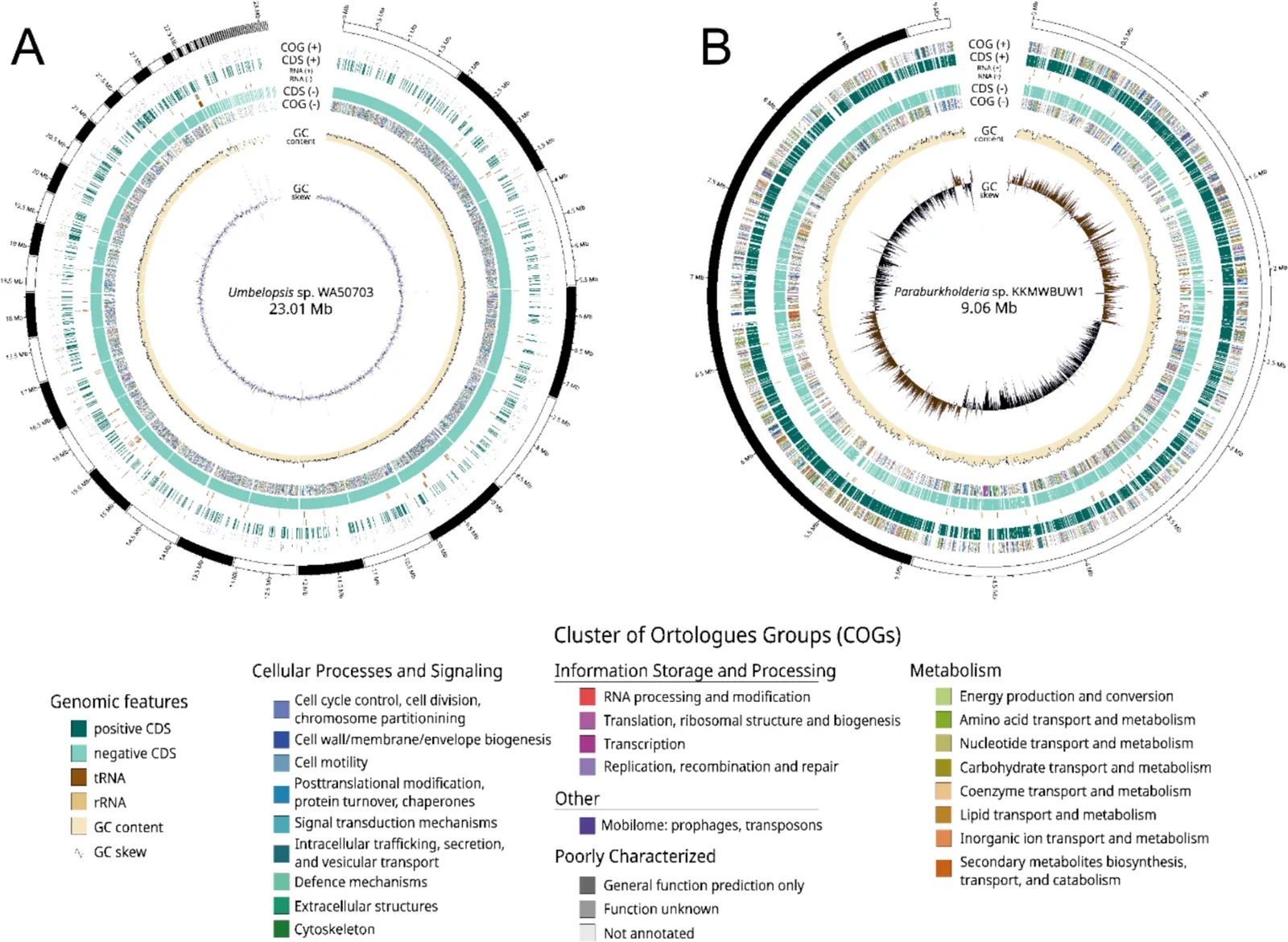
Annotated contig-level genomic assemblies of *Umbelopsis* sp. WA0000050703 (**A**) and *Paraburkholderia* sp. KKMWBUW1 (**B**) visualized using GenoVi v0.4.3 [59]

Long-read-only flye assembly was polished with Illumina reads using polypolish. After discarding contigs shorter than 1000 nucleotides. We obtained 69 contigs with a total length of 23 007 829 nucleotides (~ 23.01 Mb; Fig. 2A) and average coverage of 25X from Nanopore sequencing and 153X from Illumina sequencing. N50 was 980 051 and L50 was 8. GC content in the final assembly was 42.08%. All of these measures are similar to the ones we obtained for other *Umbelopsis* strains in our previous work [58] and for other genomes available in the NCBI database (Table 1). Genome completeness was estimated using single copy fungal orthologous genes via BUSCO v5.8.2 with mucoromycota_odb10 database to be 96.8% complete, with 31 BUSCOs duplicated, 5 fragmented, and 46 missing. Using the Funannotate pipeline, we identified 9418 putative genes (CDS) and 154 tRNAs. Funannotate annotations for all fungal strains used in this study can be found in Additional file 3.

### Basic characteristics of the *Paraburkholderia* genome

DNA extracted from axenic culture of *Paraburkholderia* was also sequenced on both MinION and Illumina platforms. From MinION Nanopore sequencing 985 138 reads were obtained with median length of 4510 bases and median PHRED score of 8.52, which resulted in 6 567 435 000 bases sequenced. After guppy basecalling, 688 055 reads (ca. 71%) were retained with a median length of 4820 bases and median PHRED score of 9.366, which resulted in 4 918 907 000 (ca. 75%) bases passing the basecalling process. Illumina MiSeq sequencing resulted in 3 845 107 paired-end reads and after initial processing which included adapter removal and quality-based trimming, 3 796 743 paired-end reads were retained.

As the average long-read sequencing coverage was very high, after testing and evaluating a few different combinations of datasets, assemblers, and polishing tools (see Methods section), we decided to use only MinION data to assemble and polish bacterial genome. Our approach resulted in two long contigs (5 and 3.9 Mb) with an average coverage of 305x. There were also two shorter contigs in the assembly: one 270 kb probable plasmid and 3 kb fragment of phage origin. For downstream analysis we used three longest contigs and discarded the shortest one due to its small size and exclusively non-bacterial annotations. Final assembly had the length of 9 063 050 bp (see Fig. 2B), which is slightly more than most free-living *Paraburkholderia* (see Table 2, Fig. 4). Our assembly is probably at a chromosomal level as genome assemblies of closely related bacterial strains (e.g. *Paraburkholderia phytofirmans* PsJN and *Paraburkholderia* sp. OLGA-172) have similar lengths of contigs and sizes of whole assemblies. The GC content in the final assembly was 61.3% and coding density was 86.6%. In the assembled genome we identified 8085 CDS, 18rRNAs and 68 tRNAs. All of these features are similar to the ones of the genomes of free-living *Paraburkholderia* strains (Table 2). BUSCO analysis confirms that our assembly is complete: there are no missing BUSCOs, and of 569 BUSCO groups present in betaproteobacteria_odb10, 563 are complete and single copy, 5 are complete and duplicated, and one is fragmented.

Genomic annotations for all bacterial strains used in this study can be found in Additional file 4.

### Comparative genomics of *Umbelopsis*

**Fig. 3:**
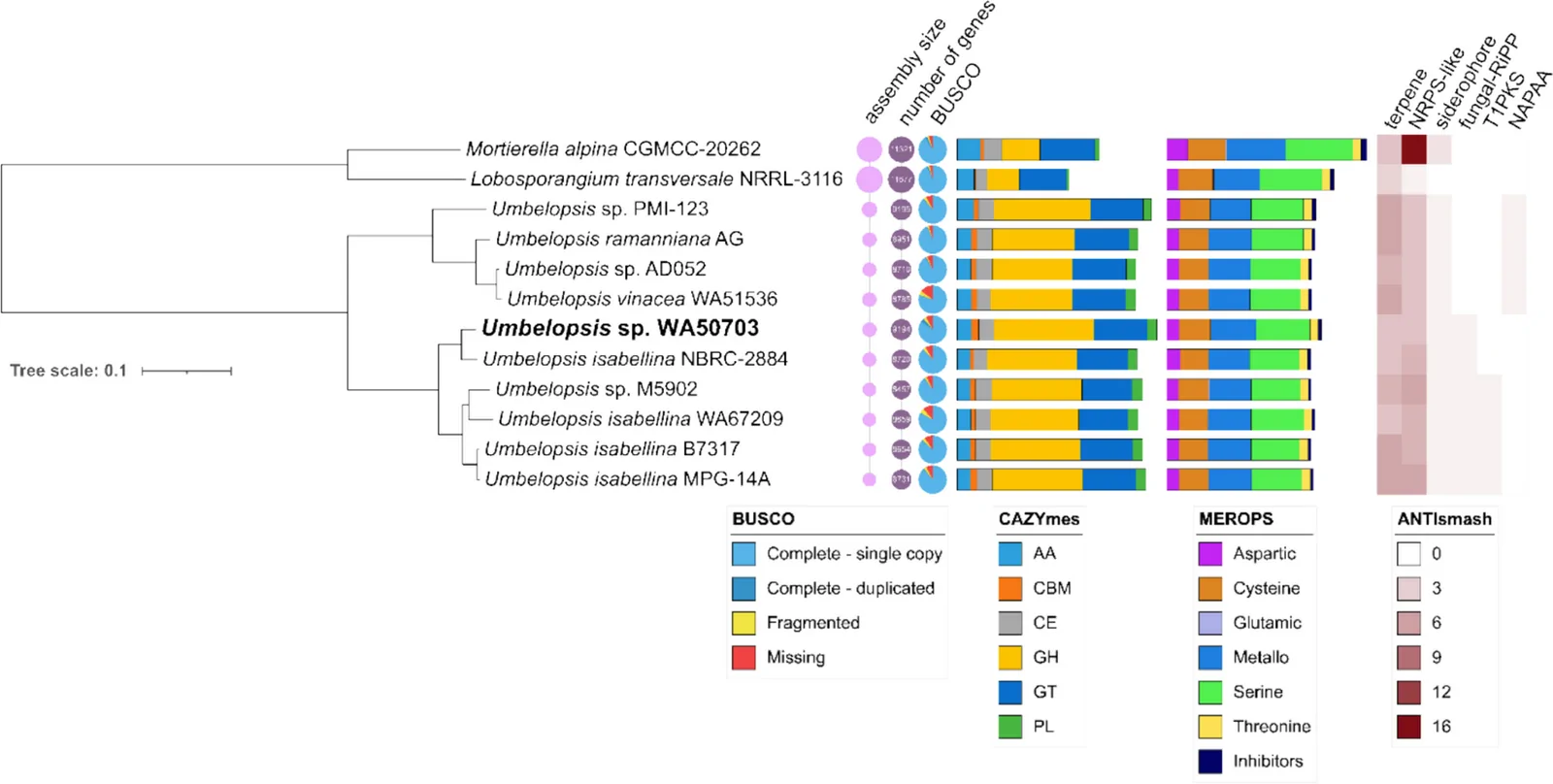
Annotated phylogenomic tree of predicted proteomes of *Umbelopsis* spp. available in the NCBI database, with two proteomes belonging to fungi from the *Mortierellaceae* family used as an outgroup. Proteomes were predicted using funannotate pipeline and the tree itself is based on multi-sequence alignment of 1475 single-copy orthogroups (822 989 sites) and calculated using Orthofinder [51], as described in the Methods section. All nodes were supported by 100% bootstrap values. Tree annotations were added using iTOL [55]

The Orthofinder species tree was calculated using concatenated alignment of 1475 single-copy orthogroups from the 13 predicted fungal proteomes (Fig. 3). Concatenated multi-sequence alignment had 822 989 sites. Based on the tree, *Umbelopsis* strain WA0000050703 is placed in the *Umbelopsis isabellina* section (as defined by Gęsiorska et al. in prep.). Querying UNITE database [60] shows that the strain belongs to SH1461563.10FU (classified as *U. dimorpha*) at 0.5% threshold value, grouping together with 1704 other ITS rDNA sequences with global distribution. At a 1% threshold value, it belongs to SH1063795.10FU (classified as *U. dimorpha*) and at 1.5% threshold to SH0742454.10FU (classified as *U. isabellina*), grouping with 1845 and 3692 other ITS rDNA sequences, respectively, also with global distributions. As phylogeny of the *Umbelopsis* genus remains largely unresolved [18], we refrain from using species name for the strain used in this study.

The genome of *Umbelopsis* WA0000050703 resembles those of other *Umbelopsis* species in the majority of analyses we performed. Genome size, number of predicted genes and level of completeness are similar across the whole genus. One of the features distinguishing WA0000050703 from other *Umbelopsis,* is the slightly higher number of predicted CAZymes, which is mostly due to higher number of glycoside hydrolases (Fig. 3). Our strain, similarly to other tested strains (see Additional file 5, CAZymes), harbored genes encoding enzymes potentially involved the decomposition of dead plant matter, such as endoglucanases (GH8, GH9, GH12), acetyl xylan esterases targeting glucuroxylans (GH67, CE2, CE4), β-glucosidases (GH1, GH3), and predominantly bacterial family of β-xylosidases (GH52). We also detected some CAZymes targeting chitin and chitosan, thus responsible for fungal cell wall remodeling, such as endochitinases (GH18) and N-acetyl β-glucosaminidases (GH20), as well as chitin synthases (GT2), α−1,2-mannosyltransferases (GT15), β−1,3-Nacetylglucosaminyltransferases/β−1,3-glucuronyltransferases (GT49), and chitooligosaccharide deacetylases (CE4). We also detected α-L-fucosidase (GH95) which targets fucose, another component of the cell wall of *Umbelopsis* [61]. There are also a few CAZyme families which probably enable degradation of fungal glucans (GH5, GH17, GH71, GH81, GH152), and galactomannans (GH27). Finally, there were also genes encoding glycohydrolase families which target bacterial peptidoglycan, such as lysozymes (GH24, GH25). While genes putatively encoding majority of these enzymes were present in all of the tested *Umbelopsis* strains (see Additional file 5, CAZymes), WA0000050703 had the highest number of GH18 copies (21 compared to avg. 15 in other strains).

*Umbelopsis* WA0000050703 also has the highest number of predicted proteases out of the tested *Umbelopsis* proteomes (Fig. 3), which is mostly due to slightly higher number of predicted metalloproteases (96 compared to average 88) and serine proteases (113 compared to average 106, see Additional file 5, MEROPS). However, all studied *Umbelopsis* strains have similar proteases repertoire which differs from *Mortierellaceae*. WA0000050703 has the lowest number of predicted secondary metabolite clusters out of tested *Umbelopsis* strains, including four terpene clusters, four NRPS-like, one siderophore, and one fungal RiPP (Fig. 3, Additional file 5, SecMet).

In the fungal genome in question, similarly to the ones analyzed previously (28), we identified multiple copies of gene encoding elongation of fatty acids protein 2 (ELO2, Additional file 5, Lipids), which is a component of elongase 2 and is responsible for biosynthesis of up to 22-carbon very long chain fatty acids [62]. Fatty acid desaturation is a next step in producing polyunsaturated fatty acids (PUFAs), of which *Umbelopsis* representatives are efficient producers [63]. Similarly to our previous findings [64], in the genome of *Umbelopsis*, we found genes encoding acyl CoA desaturase 1 Ole1, which allows for palmitoleic acid synthesis, and three desaturases (D-9, D-12, D-6), as well as MAW3 desaturase (Additional file 5, Lipids). Together these enzymes allow for synthesis of alpha-linoleic acid (18:3, n-3), and stearidonic acid (18:4, n-3). In the genome of *Umbelopsis* WA0000050703 we did not find genes encoding delta-5 desaturase, which would potentially allow for production of one of the most industrially valuable fatty acids—arachidonic acid (C20, 4n-6). These results are in agreement with in vivo studies which repeatedly show that members of the *Umbelopsis* genus can only synthesize PUFAs with up to 18 carbon chains [63, 65].

### Comparative genomics of *Paraburkholderia*

From the genome assembly we retrieved six copies of the 16S rRNA gene sequence. Sequences were almost identical (over 99.6% similarity) and when blasted against the NCBI nucleotide database, resulted in high quality hits (e-value = 0) to the 16S rRNA sequences obtained via PCR and Sanger sequencing of the *Paraburkholderia* strain KKMWBUW1, from our previous study [28] (NCBI accession numbers: MW055797-MW055801). Based on whole genome skani calculations (Additional file 1, Fig. AF2), *Paraburkholderia* KKMWBUW1 probably represents a novel species most closely related to *P. madseniana* RP11 (90.7%) and *P. caffeinilytica* CF1 (90.4%).

Comparison of the basic genomic characteristics between *Paraburkholderia* strain KKMWBUW1 and 16 free-living and endohyphal *Burkholderiaceae* strains shows higher similarity of the strain in question with other free living *Paraburkholderia* spp. than with other endohyphal bacteria from the *Burkholderiaceae* family. The genome of KKMWBUW1, in contrast to the genomes of endohyphal *Burkholderiaceae* (*Candidatus* Glomeribacter gigasporarum, *Mycetohabitans* spp., and *Mycoavidus* spp.), is non-reduced when compared to its free living relatives.

**Fig. 4:**
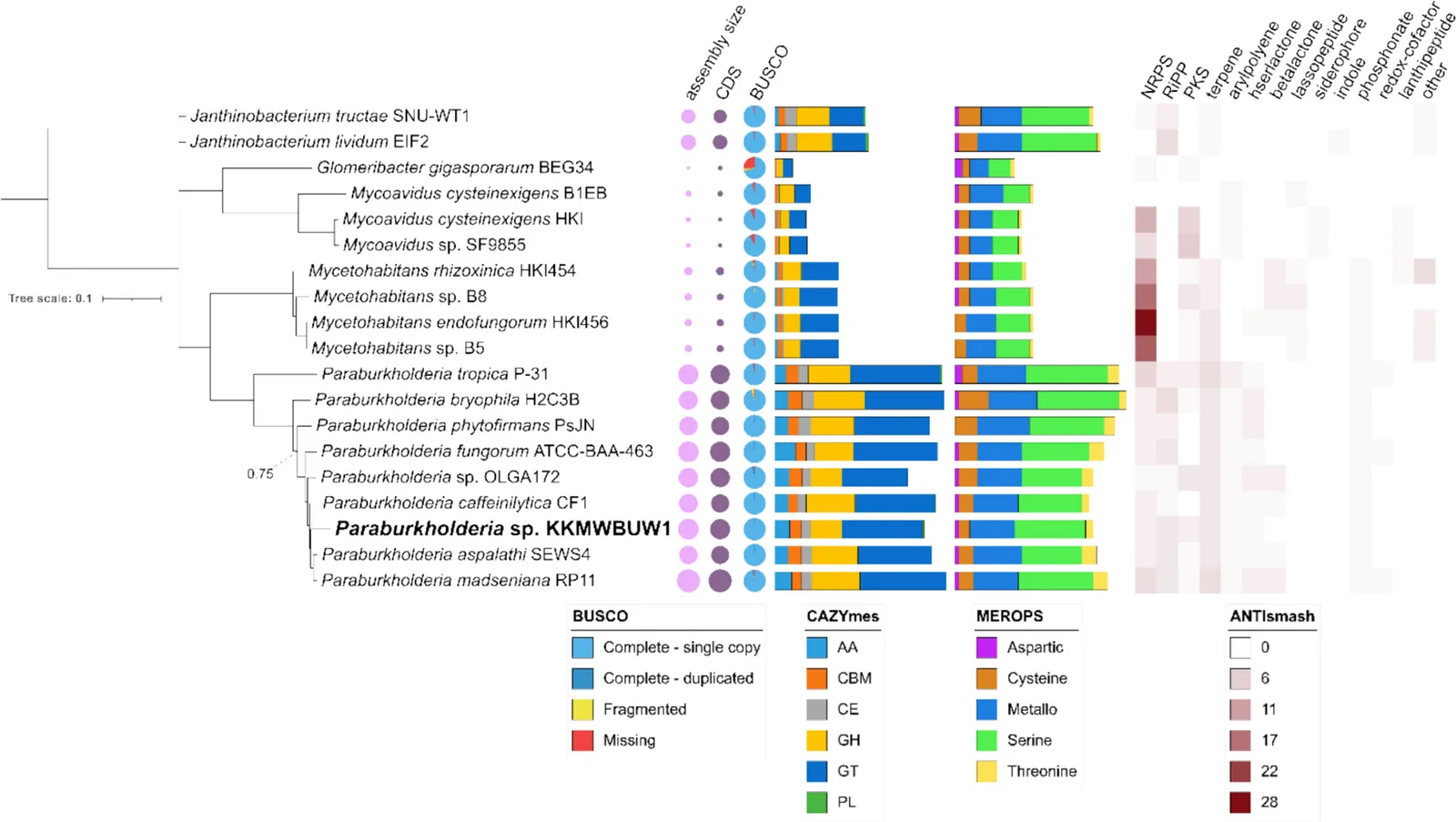
Annotated phylogenomic tree of predicted proteomes of chosen free-living and endohyphal *Burkholderiaceae* spp. available in the NCBI database, with two proteomes belonging to bacteria from the *Janthinobacterium* genus used as an outgroup. Proteomes were predicted using bakta and the tree itself is based on multi-sequence alignment of 656 single-copy orthogroups (220 850 sites) and calculated using Orthofinder [51]. All but one (marked with its value) nodes were supported by 100% bootstrap values. Tree annotations were added using iTOL [55]

Genome completeness and gene number of the strain is also very similar to other free living *Paraburkholderia*, and strikingly different from BRE. A similar pattern is observed in the predicted enzymatic capabilities of these bacteria, as both average number of CAZymes and peptidases is much higher (3 × and 2x, respectively, see Fig. 4) in free-living *Burkholderiaceae* than in BRE.

A strikingly different pattern is visible for secondary metabolite clusters, which are much more abundant in BRE (and especially *Mycetohabitans* spp.) than in free-living *Burkholderiaceae*. This difference is especially pronounced in NRPS and NRPS-like metabolite clusters (Fig. 4).

### Hydrolytic enzymes of *Paraburkholderia*

In the KKMWBUW1 genome, we identified genes encoding 156 different CAZyme families, which is a number similar to analyzed free-living *Paraburkholderia* (139–179, avg. 164), and much higher than endohyphal *Burkholderiaceae* (19–67, avg. 49). While the potential CAZymes repertoire of the strain studied here is similar to other free-living *Burkholderiaceae*, the biggest difference can be seen in the lower number of genes encoding glycoside hydrolases—GH (Fig. 4, Additional file 6). The genome of KKMWBUW1 encodes 32 different GH families, while other free-living strains range from 40 to 53 (with the exception of *Paraburkholderia* sp. OLGA-172 with the same number of KKMWBUW1). Moreover, only ca. 21% of the CAZymes-encoding genes in the genome of KKMWBUW1 are encoding glycoside hydrolases, which is lower than both free-living *Paraburkholderia* (23–30%, avg. 27%), and BRE (24–42%, avg. 30%).

In the genome of *Paraburkholderia* strain KKMWBUW1 we found more copies (12) of genes encoding AA3_2 than in free-living *Burkholderiaceae* (5–8, avg. 6.25) and in endohyphal strains (0). Enzymes from this family are abundant in wood-degrading fungi [66] and their main function is the stimulation of lignocellulose degradation in cooperation with other enzymes, such as peroxidases from the AA2 family [67], which are encoded by the genomes of both *Umbelopsis* WA0000050703 and *Paraburkholderia* KKMWBUW1, as well as all other free-living *Paraburkholderia* studied here, and none of the BRE.

**Fig. 5:**
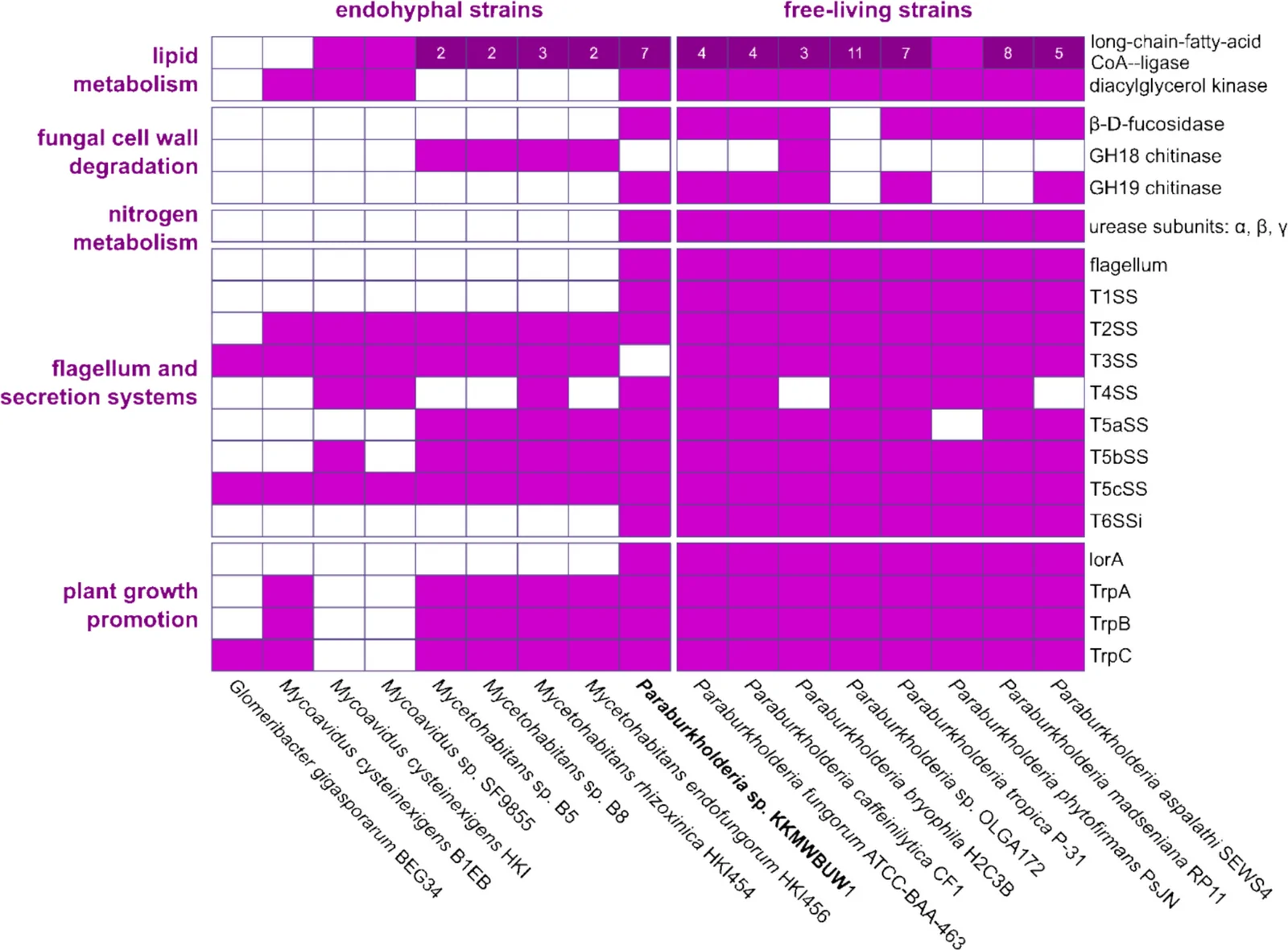
Comparison of genomic features between free-living and endohyphal *Burkholderiaceae*. The first row shows the number of copies of the gene encoding long-chain-fatty-acid-CoA ligase present in each genome, and the rest is presence/absence data

In the genome of *Paraburkholderia* strain KKMWBUW1 we identified genes putatively encoding enzymes from glycoside hydrolases family 19 (GH19). These enzymes are referred to as endochitinases (EC 3.2.1.14) as they can split chitin polymers into N-acetyl-D-glucosamine (GLcNAc) oligomers, as well as facilitate further chitin degradation into GlcNAc monomers [68]. Enzymes from this family seem to be less effective in chitin degradation than GH18 chitinases (identified in *Umbelopsis*), however they are able to degrade chitosan, which is one of the main components of *Mucoromycota* fungal cell wall [61]. Genes encoding GH19 were not present in any of the other tested endohyphal strains, whereas GH18 were detected in all *Mycetohabitans* strains (see Fig. 5).

We also identified a gene encoding bifunctional protein β-glucosidase (EC 3.2.1.21)/β-D-fucosidase (EC 3.2.1.38) in the genome of *Paraburkholderia* strain KKMWBUW1 (CAZy: GH1_e107, Additional file 6). This enzyme has a wider activity spectrum, as, apart from fucose and glucose, it also allows for beta-glucan and polyphenol degradation. It was present in all but one (*Paraburkholderia* strain OLGA172) analyzed genomes of free living *Burkholderiaceae* (see Fig. 5). It was however not present in any of the analyzed endohyphal bacterial genomes. As *Umbelopsis* spp. seem to have over twice to three fold more fucose in their cell walls when compared to *Muco*r spp [58]. and high amounts of this compound in the cell wall is considered a distinct feature of *Mucoromycota* fungi [69], the presence of the genes encoding enzymes responsible for its degradation in associated bacteria is of particular interest.

### Secretion systems of *Paraburkholderia*

*Paraburkholderia* strain KKMWBUW1 has all the genes encoding for proteins from type I secretion system. Its genome also encodes almost all proteins from type II secretion system (T2SS, General Secretion Pathway) machinery. We did not identify any genes encoding proteins from T3SS. T4SS seems in turn almost complete, however we did not find genes encoding pilus (*virB2*), one of two surface proteins (*virB3*), and one outer membrane protein (*virB7*) (Additional file 1, Fig. AF3).

*Paraburkholderia* strain KKMWBUW1 has three sets of genes encoding type VI secretion system proteins, one of them present on the third contig, which is possibly a plasmid. The plasmid placement is in agreement with a recent study [70]. T6SS is a syringe-type molecular mechanism which enables bacteria to interact with adjacent cells by injecting specific proteins into their cytoplasm or surrounding environment [71–73]. In the genome of *Paraburkholderia* strain KKMWBUW1 we identified all necessary components of T6SS subtype i (T6SS^i^), which is a subtype typically found in Proteobacteria [74].

## Discussion

Unlike other studied interactions between *Mucoromycota* and *Burkholderiaceae*, the interaction characterized here was demonstrated to be distinctly facultative. While our initial hypothesis suggested a transient association based on niche proximity, our results demonstrate an actively maintained relationship. The ability of *Paraburkholderia* strain KKMWBUW1 to grow axenically, while remaining present within fungal hyphae and vertically transmitted through spores, indicates a stable but non-obligate endohyphal lifestyle. This is further supported by the lack of genome reduction in either partner, typical for obligate endosymbiosis, aligning instead with facultative EHB recently isolated from diverse *Ascomycota* [75]. The genomic stability of both partners suggests that the capacity for this interaction may rely on default genetic machinery rather than specialized symbiotic acquisitions.

In recent years, there is growing evidence that fungi, similarly to other groups of organisms (like animals and plants) should be considered holobionts [76], interacting with various epihyphal and endohyphal bacteria (e.g. [5]). However, BFI are way less studied than for example animals [77] and therefore interpretation of results is more complicated. In majority of mycological research, presence of interacting bacteria is usually not verified and not reported in results. Moreover, bacterial sequences are usually removed from genomes during analysis as contamination. Together, these two aspects constrain our current understanding of the factors underlying why certain fungi can serve as hosts for bacteria whereas others cannot. Another explanation for lack of specific genomic traits that could characterize BFI is that BFI studies are not very advanced yet and there is also probability that we did not find genes involved in maintaining facultative interactions as they have not been characterized yet. Even though the genomes of both partners are non-reduced and similar to their free-living relatives, there are several elements of genomic content of both partners that may be related to the interactions of both partners.

We identified genes for long-chain-fatty-acid–CoA ligase (*fadD* in bacteria, *faa* in fungi) in both genomes, suggesting that *Paraburkholderia* may metabolize host-produced fatty acids, similar to *Mycoavidus cysteinexigens* [10]. However, we identified homologs of these genes in all free-living strains and all but one (*Candidatus* Glomeribacter gigasporarum BEG34) endohyphal strains.

Furthermore, the presence of diacylglycerol kinases (DGK) in both partners is noteworthy. In the *Rhizopus-Mycetohabitans* system, shifts in lipid ratios via DGK activity determine the transition from mutualism to antagonism [78]. The widespread presence of DGK across our tested strains suggests its role in fungal-bacterial interactions (BFI) warrants broader investigation as a potential regulatory switch.

Nitrogen and carbon metabolism also suggest the collaborative nature of this wood-decay consortium. While *Paraburkholderia* KKMWBUW1 lacks the key nitrogenase gene (*nifH)* for nitrogen fixation, the presence of accessory genes (*nifQ, nifE*) together with the fact that closely related bacteria can fix nitrogen, make nitrogen metabolism in KKMWBUW1 worth further investigation. Additionally, the presence of genes encoding all three subunits (alpha, beta, gamma) of urease enzyme, together with genes encoding urea ABC transporters (UrtABCDE) and urease accessory proteins (UreDEFG) in *Paraburkholderia* strain KKMWBUW1 genome suggest that the consortium can use urea as a nitrogen source by hydrolyzing it to ammonia in low-nitrogen environments.

Regarding carbon acquisition, fungus seems to be able to produce peroxidases as well as endoglucanases and β-glucosidases which may be responsible for initial steps of plant lignocellulolytic degradation, whereas the bacteria encode fewer such enzymes. This disparity suggests a nutritional crosstalk where the bacteria rely on the fungus to break down plant polysaccharides, while potentially contributing to the degradation of pectins, catechols, phenols, sodium benzoate, phenyl acetate, and furfural, which is a natural dehydration product of xylose, a pentose sugar often found in large quantities in the hemicellulose fraction of lignocellulosic biomass [79]. It is also worth mentioning that *Umbelopsis* WA0000050703 has more a more diverse repertoire of glycoside hydrolases than other *Umbelopsis* strains, while genome of *Paraburkholderia* KKMWBUW1 seems to encode less glycoside hydrolases than other *Paraburkholderia* strains. As the consortium was isolated from deadwood these characters may be related to specific niche adaptation.

Mechanistically, the *Umbelopsis-Paraburkholderia* interaction appears less manipulative than its obligate counterparts. The presence of the Type II Secretion System (T2SS), linked to the secretion of cell-wall-degrading enzymes in *Rhizopus* symbionts [8], likely facilitates bacterial entry into the hyphae [80]. However, the absence the Type III Secretion System (T3SS), which is essential for host manipulation in obligate relationships, reinforces the hypothesis on facultative nature of this association.

Ecologically, the presence of plant growth-promoting (PGP) traits, such as indolepyruvate oxidoreductase and phosphorus mineralization capacity, suggests the consortium can play a dual role in the forest ecosystem. Given that *Umbelopsis* is frequently isolated from both living roots [19, 26, 27] and decaying wood wood in the late stages of decay [22, 23], we hypothesize that this consortium functions as a fungal holobiont that transitions from an endophytic to a saprotrophic lifestyle. In the late stages of wood decay, the consortium may facilitate forest regeneration by promoting the growth of seedlings on nurse logs. This study provides a foundational starting point for exploring facultative EHB interactions, representing a significant shift from the obligate models that have historically dominated BFI research.

Further studies of *Umbelopsis-Paraburkholderia* interaction can provide new insights into the biology of interactions between fungi and endohyphal bacteria as it represents a different type of symbiosis from EHB studied so far. Genomic and microscopic characterization of the consortium provides a starting point for further exploration of the nature of this interaction and its role in the ecosystem.

## Conclusions

As studying bacterial-fungal interactions became feasible relatively recently, not much is known about their biology and ecology. Most studies so far focused on strict and obligatory pairs with clear impact of partners on each other. In this study we focused on a facultative relationship between *Umbelopsis* sp. and *Paraburkholderia* sp., both close relatives to organisms forming obligatory partnerships with each other. We proved that even though the relationship is facultative and that EHB presents a metabolic cost to its partner, bacteria is present within fungal hyphae and spores. *Paraburkholderia*, unlike strictly endohyphal symbionts, has a non-reduced genome, with extensive metabolic capacities and a complete molecular apparatus needed for establishment and maintenance of endohyphal interaction with their host. While we refrain from forming any strong conclusions without experimental confirmation, it is probable that it is *Paraburkholderia* that enables *Umbelopsis* to occupy otherwise unavailable niches, such as decaying wood.

## Supplementary Information


Additional file 1: Additional figures, captions included in the file
Additional file 2: Raw data used for growth plots
Additional file 3: Funannotate annotations for all fungal strains used in this study
Additional file 4: Bakta annotations for all bacterial strains used in this study
Additional file 5: Detailed annotations of CAZYmes, peptidases, secondary metabolite clusters, and genes involved in lipid metabolism for WA0000050703, as well as comparisons with all other fungal strains used in this study
Additional file 6: Detailed annotations of CAZYmes for all bacterial strains used in this study


## Data Availability

Fungal strain is deposited in the Herbarium of the University of Warsaw, and bacteria in the Culture Collection of the University of Warsaw. Genomic datasets supporting the conclusions of this article are available in the NCBI Repository: BioProject PRJNA1042639, while transcriptomic dataset can be accessed in the ENA Repository: Project PRJEB98689.

## Abbreviations

BFI: Bacterial-fungal interactions
BRE: *Burkholderia-*related endohyphal bacteria
DGK: Diacylglycerol kinase
EHB: Endohyphal bacteria
MRE: *Mollicutes/Mycoplasmataceae*-related endohyphal bacteria
PDA: Potato dextrose agar
PDB: Potato dextrose broth
PRE: *Paraburkholderia-*related endohyphal bacteria
TxSS: Type X Secretion System
CAZYmes: Carbohydrate-Active enZYmes
GH: Glycoside Hydrolases
GT: Glycosyl Transferases
PL: Polysaccharide Lyases
CE: Carbohydrate Esterases
AA: Auxiliary Activities
