# Supplementary material for: Characterization of facultative yet endohyphal interaction between Umbelopsis and Paraburkholderia
Source: BMC Genomics. 2026 Jun 19;27:718. doi: 10.1186/s12864-026-13055-5 (PMC13528069; doi:10.1186/s12864-026-13055-5)
Supplement: Supplementary file 1 [file 12864_2026_13055_MOESM1_ESM.docx]

# Additional file 1: Figures A1-A3

Figure A1. Growth curves for *Umbelopsis* WA0000050703 with (orange) and without (blue) endohyphal *Paraburkholderia* KKMWBUW1 in (A) rich and (B) minimal liquid media.

Figure A2. Graph showing whole genome skani calculations of all bacterial strains used in this study in relation to *Paraburkholderia* KKMWBUW1. Values are percentages of similarity to the genome of KKMWBUW1.

Figure A3. KEGG map of bacterial secretion systems. The proteins marked in blue were found in predicted proteome of *Paraburkholderia* KKMWBUW1, in green are proteins found in both *Paraburkholderia* KKMWBUW1 and *Umbelopsis* WA0000050703. Proteins with no highlight were not found in any of the proteomes.
